# Enhanced Photocatalytic Activity of Nanoparticle-Aggregated Ag–AgX(X = Cl, Br)@TiO_2_ Microspheres Under Visible Light

**DOI:** 10.1007/s40820-017-0150-8

**Published:** 2017-07-19

**Authors:** Cuiling Zhang, Hao Hua, Jianlin Liu, Xiangyu Han, Qipeng Liu, Zidong Wei, Chengbin Shao, Chenguo Hu

**Affiliations:** 1 Chongqing Engineering Laboratory for Detection, Control and Integrated System, Chongqing Technology and Business University, Chongqing, 400067 People’s Republic of China; 2 College of Chemistry and Chemical Engineering, College of Physics, Chongqing University, Chongqing, 400044 People’s Republic of China

**Keywords:** Ag–AgX(X = Cl, Br)@TiO_2_, Nanoparticle-aggregated spheres, Methyl orange, Visible light, Photocatalysis

## Abstract

Ag–AgX(X = Cl, Br)@TiO_2_ nanoparticle-aggregated spheres with different mass ratio of *R* = TiO_2_/Ag(X) from 35:1 to 5:1 were synthesized by a facile sol–gel technique with post-photoreduction. The photocatalytic activities of both Ag–AgCl@TiO_2_ and Ag–AgBr@TiO_2_ under visible light are effectively improved by ~3 times relative to TiO_2_ NPAS under the simulated sunlight for the decomposition of methyl orange (MO). Ag–AgBr@TiO_2_ showed 30% improvement and less stable in photocatalytic activity than that of AgCl@TiO_2_. The role of Ag and AgX nanoparticles on the surface of Ag–AgX(X = Cl, Br)@TiO_2_ was discussed. Ag on these samples not only can efficiently harvest visible light especially for AgCl, but also efficiently separate excited electrons and holes via the fast electron transfer from AgX(X = Cl, Br) to metal Ag nanoparticles and then to TiO_2_-aggregated spheres on the surface of heterostructure. On the basis of their efficient and stable photocatalytic activities under visible-light irradiation, these photocatalysts could be widely used for degradation of organic pollutants in aqueous solution.

**Electronic supplementary material:**

The online version of this article (doi:10.1007/s40820-017-0150-8) contains supplementary material, which is available to authorized users.

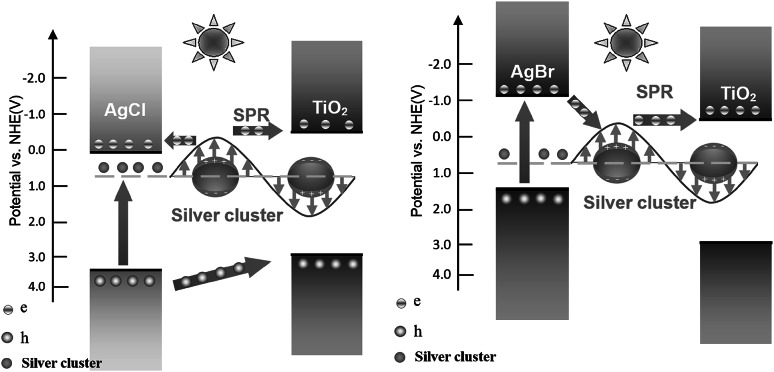

## Highlights

- Ag–AgX(X = Cl, Br)@TiO_2_ nanoparticle-aggregated spheres (NPAS) have been designed as a photocatalyst, in which AgX works as light harvester, Ag conducts as electron trapping and accumulating site, and TiO_2_ acts as electron collecting and photocatalytic site.
- The photocatalytic activities of Ag–AgX(X = Cl, Br)@TiO_2_ by degradation of methyl orange (MO) under visible light were improved by ~3 times relative to TiO_2_ NPAS under simulated sunlight.

## Introduction

As an important photocatalyst, titanium dioxide (TiO_2_) has been widely used in decomposition of toxic and hazardous organic pollutants, and in water splitting for hydrogen production [1–3]. However, the rapid recombination of photoinduced electrons and holes and its wide band gap greatly lower the TiO_2_ quantum efficiency. Therefore, it is of great importance to improve the visible-light harvesting and the separation of photoinduced electron–hole pairs in TiO_2_ for its further applications. TiO_2_-based heterostructures or composites formed by semiconductors with narrower band gap (e.g. Ag_2_O/TiO_2_, BiVO_4_/TiO_2_/BiVO_4_, BiOI/TiO_2_, Cu_2_O/TiO_2_, and Bi_2_O_3_/TiO_2_) have been intensively investigated to develop high activities [4–8]. In addition, noble metal–TiO_2_ is used to improve the generation and separation of photoinduced carriers due to the surface plasmon resonance (SPR) of noble metal nanoparticles such as Ag and Pd [9–12]. The photoinduced electrons in noble metal transfer onto the surface of semiconductor, which results in visible-light photocatalytic activity, such as Ag@TiO_2_ or Ag nanoparticles and graphene-co-loaded TiO_2_. Therefore, noble metal–semiconductor junction has potential application for the visible-light response in environmental purification. Specifically, because noble metal has a strong electron storage property and large work function, the excited electrons transfer from TiO_2_ to a noble metal nanoparticle is an ultrafast process [9]. This phenomenon improves photocatalytic activity by effective charge separation. However, there are two opposite electron transfers in the metal–semiconductor structure which is from noble metal to semiconductor and from semiconductor to noble metal, respectively, under an irradiation. Thus, it is important to steer the transfer of the photoinduced carriers.

In recent years, AgX [13–22] and Ag/AgX(X = Cl, Br) have been paid much interest in the design of photosensitive composite materials because of their strong absorption of visible light by their surface plasmon resonance (SPR) and self-sensitization. It provides new opportunity for developing visible-light-driven photocatalyst. For example, it has been demonstrated that Ag/AgCl–BiOX (X = Cl, Br) [16], AgI/AgCl/TiO_2_ [17], and 3D AgX/graphene aerogels (X = Br, Cl) [18], AgCl_*x*_Br_1−*x*_ nanocrystals [19], AgBr supported on g-C_3_N_4_-decorated nitrogen-doped graphene ternary nanocomposites [20] and Ag–AgBr/TiO_2_ composite [21] showed enhanced photocatalytic activities. In addition, the morphology of photocatalyst is important for the photocatalytic activity [23–28]. The pristine TiO_2_ nanoparticles-aggregated spheres (NPAS), Ag_2_O/TiO_2_ NPAS, and Pt/TiO_2_ NPAS showed excellent photocatalytic activity due to their large surface area and high adsorption of dyes [8, 24]. Meanwhile, because of its different band structures, AgX(X = Cl, Br) shows different photoresponsive behavior under visible light and the degradation mechanism of AgX(X = Cl, Br)/TiO_2_ on methyl orange (MO) might be different.

Herein, we developed a facile low-temperature method to prepare Ag–AgX(X = Cl, Br)@TiO_2_ NPAS heterojunction photocatalysts and the influence of weight rate *R* (R = TiO_2_:AgX) and band structures of heterojunctions on the photocatalytic activity were systematically studied. Ag–AgX(X = Cl, Br)@TiO_2_ double heterostructures exhibited much higher photocatalytic activity for the degradation of MO under visible-light irradiation (*λ* > 400 nm). The possible mechanism and the key role of metallic Ag on the photocatalytic activity of the Ag–AgX(X = Cl, Br)@TiO_2_ double heterostructures were proposed.

## Experimental Details

### Preparation of Samples

All chemical reagents used in this experiment are of analytical purity and without further purification.

#### Preparation of Ag–AgCl@TiO_2_ NPAS

TiO_2_ NPAS were prepared by diglycol-mediated process with minor modification, which was reported elsewhere [24]. For Ag–AgCl@TiO_2_ NPAS preparation, 0.23 g of TiO_2_ NPAS was dispersed in 20 mL deionized water with vigorous stirring for 1 h, while 0.15 g cetyltrimethylammonium chloride (CTAC) was added dropwise to the suspension and stirred for 1 h. Then, 800 µL 0.1 mol L^−1^ AgNO_3_ aqueous solution was added and continually stirred for 1 h and then irradiated for 30 min under the simulated sunlight. The precipitation was collected by a washing and centrifugation process, and then the obtained sample was dried at 60 °C in a drying oven for 12 h. Finally, the precursors were completely crystallized and converted into Ag–AgCl@TiO_2_ NPAS by heating at 300 °C for 8 h in nitrogen. Ag–AgCl@TiO_2_ with weight ratio (*R* = TiO_2_:AgCl) of 1:0, 30:1, 25:1; 20:1; 15:1; 10:1; 5:1 denoted as sample S1–S7 was prepared by changing the amount of TiO_2_ NPAS as shown in Table 1.

**Table 1:** Experimental condition for the preparation of different Ag–AgCl@TiO_2_ NPAS samples

Sample	TiO_2_ NPAS (g)	CTAC (g)	0.1 M AgNO_3_ aq. (µL)	*R* = TiO_2_/AgCl
S2	0.3439	0.1536	800	30:1
S3	0.2866	0.1536	800	25:1
S4	0.2293	0.1536	800	20:1
S5	0.1719	0.1536	800	15:1
S6	0.1146	0.1536	800	10:1
S7	0.0573	0.1536	800	5:1

#### Preparation of Ag–AgBr@TiO_2_ NPAS

The preparation of Ag–AgBr@TiO_2_ NPAS is similar to that of Ag–AgCl@TiO_2,_ with cetyltrimethylammonium bromide (CTAB) added rather than CTAC. The Ag–AgCl@TiO_2_ with weight ratio (*R* = TiO_2_:AgBr) of 35:1, 30:1, 25:1, 20:1, 15:1, 10:1, 5:1 is denoted as sample S8–S14 as shown in Table 2.

**Table 2:** Experimental condition for the preparation of different Ag–AgBr@TiO_2_ NPAS samples

Sample	TiO_2_ NPAS (g)	CTAB (g)	0.1 M AgNO_3_ aq. (µL)	*R* = TiO_2_/AgBr
S8	0.5257	0.1749	800	35:1
S9	0.4506	0.1749	800	30:1
S10	0.3755	0.1749	800	25:1
S11	0.3004	0.1749	800	20:1
S12	0.2253	0.1749	800	15:1
S13	0.1502	0.1749	800	10:1
S14	0.0751	0.1749	800	5:1

### Characterization

X-ray photoelectron spectroscopy (XPS) patterns of Ag–AgX@TiO_2_ NPAS were determined by photoelectron spectrometer (ESCALAB 250Xi). The morphological features of the samples were characterized by a scanning electron microscope (SEM, TESCAN VEGA 3 SBH SEM), a field emission scanning electron microscopy (FE-SEM, Nova 400 Nano SEM) and a high-resolution transmission electron microscopy (HRTEM, JEOL-4000EX). The elemental composition of the samples was recorded by energy-dispersive X-ray spectroscopy (EDS, OXFORD). UV–Vis absorption spectrum was measured by the UV–Vis spectrophotometer (UV-3600, Shimadzu) with an integrating sphere attachment. Photoluminescence (PL) measurement was obtained by an Edinburgh FLS920 fluorescence spectrometer with the excitation source of steady-state Xe900 450 W xenon lamp. All the measurement was taken at room temperature.

### Photocatalytic Degradation

Twenty milligrams of samples was added into 50 mL aqueous suspension of MO (14 mg L^−1^) in a 100 mL beaker. The suspension was magnetically stirred in dark for 30 min to establish adsorption/desorption equilibrium before illumination. An simulated sunlight instrument (CHF-XM-500 W) with a power of 100 mW cm^−2^ was used as an illumination source, and the 420 nm cutoff filter was placed on the end of light transmission tube to ensure the irradiation only in visible-light wavelength. The catalysis after degradation was collected by a washing and centrifugation process and then dried at 60 °C. In order to keep the mass of catalysis constant, some catalyst (about 2 mg) were added.

## Results and Discussion

### Crystal Structure, Morphology, and Formation Mechanism of Ag–AgX(X = Cl, Br)@TiO_2_ NPAS

**Fig. 1:**
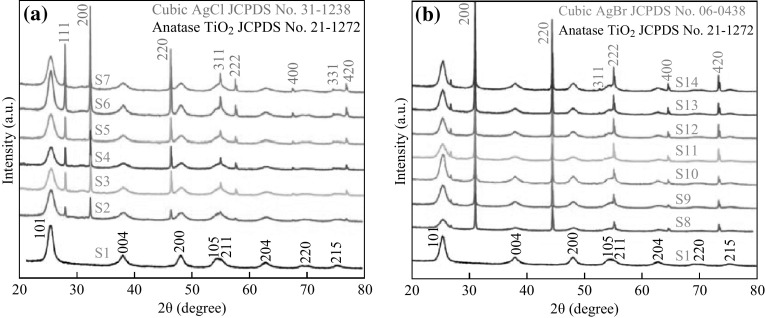
XRD patterns of **a** the Ag–AgCl@TiO_2_ NPAS samples and **b** Ag–AgBr@TiO_2_ NPAS samples

Figure 1a shows the XRD patterns of the Ag–AgCl@TiO_2_ samples S1–S7. The sample S1 with R = 1:0 exhibits pure anatase phase TiO_2_ (JCPSD No. 21-1272), while the rest of the sample S2–S7 show the diffraction peaks of pure cubic AgCl (JCPDS No. 31-1238) besides TiO_2_. Both phases of AgCl and TiO_2_ are consistent with that of the standard card. The XRD patterns of AgCl become strong from S2 to S7 with an increase of AgCl in AgCl@TiO_2_. However, obvious Ag peaks are not found in the XRD patterns in all the samples. The 2-*θ* at 38.3° corresponding to the (111) of cubic phase Ag (*F*m-3m (225), JCPDS No. 87-0719) appears to overlap with (004) (2-*θ* at 37.8°) and (112) (2-*θ* at 38.6°) of anatase phase TiO_2_, and the small pimple of 2-*θ* at 77.7° may correspond to the (311) of cubic metallic Ag. Both of them do not demonstrably show the existence of Ag for XRD patter, which demonstrates the Ag distributed on the AgCl@TiO_2_ in a very small percentage. It is because there is a little elemental Ag reduced from AgCl under illumination for 30 min [29].

Figure 1b shows the XRD patterns of the Ag–AgBr@TiO_2_ samples S8–S14. Similar to Ag–AgCl@TiO_2_, the samples show two-phase mixture of cubic AgBr (*F*m-3m (225), JCPDS No. 06-0438) and anatase phase TiO_2_ and they are all consistent with those of the standard cards. The relative intensity of AgBr to TiO_2_ increases with the decrease of R in Ag–AgBr@TiO_2_. We do not have clear evidence of Ag existence from XRD and another measurement to confirm the Ag metal should be carried out.

**Fig. 2:**
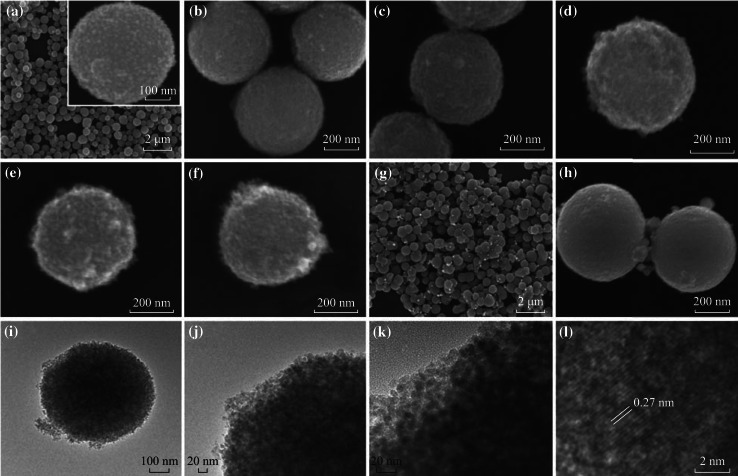
FE-SEM images of Ag–AgCl@TiO_2_ NPAS: **a** S1, **b** S2, **c** S3, **d** S4, **e** S5, **f** S6, **g**–**h** S7 and **i**–**l** TEM and HRTEM images of sample S4

**Fig. 3:**
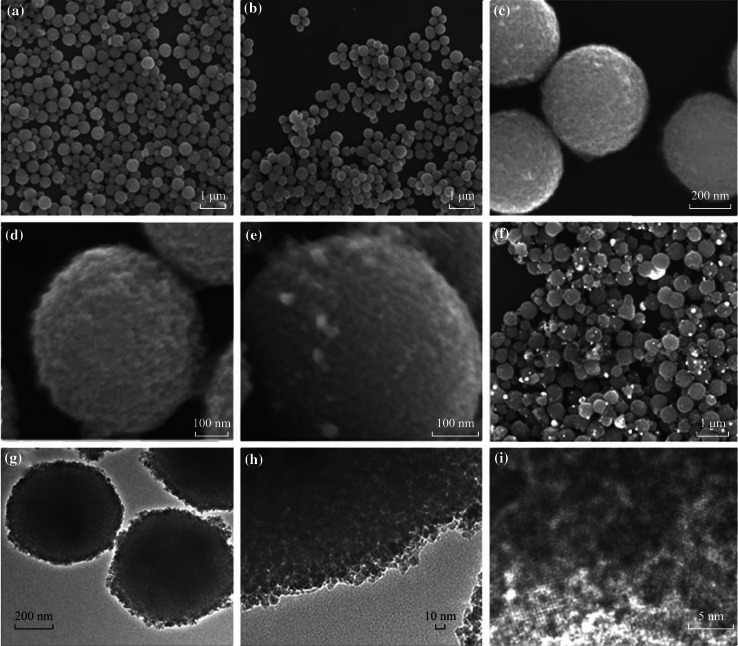
FE-SEM images of Ag–AgBr@TiO_2_ NPAS: **a** S8, **b** S9, **c** S10, **d** S12, **e** S13, **f** S14 and **g**–**i** TEM and HRTEM images of sample S11

To further obtain the structure and morphology information of Ag–AgX(X = Cl, Br)@TiO_2_ samples, FE-SEM, TEM, and HRTEM characterizations have been conducted as shown in Figs. 2 and 3. Figure 2a shows the FE-SEM and enlarged image in the inset of pristine TiO_2_ (S1) which indicates nanoparticles-aggregated spheres with the diameter of 370–450 nm. The Ag–AgCl@TiO_2_ with R from 30:1 to 5:1 (S1–S7) is shown in Fig. 2b–g, from which we can see that the diameter is about 500–600 nm with Ag–AgCl nanoparticles coated on the surface. The morphology of Ag–AgCl@TiO_2_ is similar to that of the TiO_2_-aggregated spheres, which indicates that the precipitation of Ag–AgCl on the surface of TiO_2_ does not affect the structure of TiO_2_-aggregated spheres and TiO_2_-aggregated spheres present very good sites to adsorb Ag–AgCl. With the increase of Ag–AgCl in Ag–AgCl@TiO_2_, there are more and more small particles on the surface of samples. When R = 5:1, much larger particles of Ag–AgCl appear pinned on the spheres as shown in Fig. 2g and enlarged image in Fig. 2h. No individual particles of Ag–AgCl are found, which is similar to the Ag_2_O-@TiO_2_ NPAS [24]. The TEM and HRTEM of Ag–AgCl@TiO_2_ with R = 20:1 named S4 are presented in Fig. 2i–l. The Ag–AgCl particles with a diameter of 5–15 nm are uniformly coated on the surface of the sample. These particles connect each other and the grain boundary can be seen. The perfect combination results from the lattice match of TiO_2_ and AgCl. For example, the lattice spacing of (211) (*d* = 0.167 nm) and (215) (*d* = 0.126 nm) for TiO_2_ is close to that of (311) (*d* = 0.167 nm) and (331) (*d* = 0.127 nm) of AgCl provided by the XRD patterns in Fig. 1. The lattice fringe of AgCl is observed clearly in Fig. 2l, and the resolved interplanar distance is ca. 0.27 nm, corresponding to the (200) plane e of AgCl. The EDS analysis and elemental mapping images (EMIs) of O, Ti, Ag, and Cl of Ag–AgCl@TiO_2_ are presented in Figs. S1b and S1c-f, respectively. The results reveal the uniform distributions of O, Ti, Ag, and Cl. Consequently, the Ag and AgCl nanoparticles are well distributed on the surface of TiO_2_ nanoparticles-aggregated spheres.

Figure 3a–f shows FE-SEM images of Ag–AgBr@TiO_2_ samples S8–S10 and S12–S14, respectively, from which we can see that Ag–AgBr nanoparticles are coated on the surface of TiO_2_-aggregated spheres with the diameter of 500–600 nm. The morphology of Ag–AgBr@TiO_2_ is similar to that of the TiO_2_-aggregated spheres, which indicates that the precipitation of Ag–AgBr on the surface of TiO_2_ does not affect the structure of TiO_2_ NPAS and TiO_2_ NPAS present very good sites to adsorb Ag–AgBr. With the increase of Ag–AgBr in Ag–AgBr@TiO_2_, there are more and more small particles on the surface of samples. The TEM and HRTEM images of sample S11 are presented in Fig. 3g–i. The Ag–AgBr particles about 10 nm are uniformly coated on the surface of the samples and connect each other. The corresponding EDS analysis and EMIs of Ag–AgBr@TiO_2_ are presented in Figs. S2b and S2c-f, which reveal the uniform distributions of O, Ti, Ag, and Br. Consequently, the Ag and Br are well distributed on the surface of TiO_2_ nanoparticles-aggregated spheres.

**Fig. 4:**
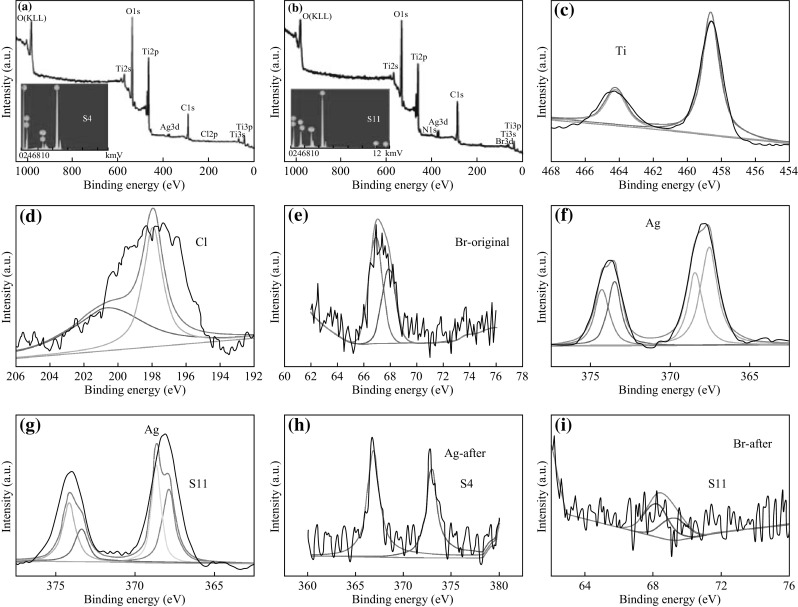
XPS patterns of sample S4. Wide survey scan for **a** Ag–AgCl@TiO_2_ and **b** Ag–AgBr@TiO_2_, **c** Ti 2*p*, **d** Cl 2*p*, **e** Br 3*p*, **f** Ag 3*d* and **g** Ag 3*d* of sample S11, **h** Ag 3*d* in S4 and **i** Br 3*p* in S11 after 5 cycles of photocatalytic degradation. The *inset* EDS patterns of samples 4 and 11 are in the *inset* in (**a**, **b**)

The surface chemical states of the Ag–AgX(X = Cl, Br)@TiO_2_ with further investigation by XPS are shown in Fig. 4. The peak positions in all the XPS spectra were calibrated with C 1s at 286.4 eV. The XPS spectrum of the Ag–AgX(X = Cl, Br)@TiO_2_ indicates that the product consists of Ag, Ti, O, and X (X = Cl, Br) elements (Fig. 4). The peak of N 1s results from the surface adsorption of nitrogen during preparation of Ag–AgX(X = Cl, Br)@TiO_2_ at 300 °C for 8 h under the protection of nitrogen. Two strong peaks at 464.3 and 458.6 eV are attributed to Ti 2*p* of TiO_2_ (Fig. 4c), which is slightly shifted toward lower binding energies compared with that of pristine TiO_2_ due to the direct contact between Ag nanoparticles and TiO_2_ [21]. The peak of Cl can be divided into two peaks Cl 2*p*_3_ and Cl 2*p*_1_ peaks (Fig. 4d) [19], which correspond to 200.5 and 197.6 eV, respectively. Br 3*d*_5/2_ and Br 3*d*_3/2_ binding energies in XPS spectra correspond to 67.8 and 68.8 eV, respectively, which could be assigned to Br^−^ in AgBr (Fig. 4e) [18]. There are two strong peaks for Ag 3*d* at about 374 and 367.8 eV as shown in Fig. 4f–g. The peaks at 374 and 367.8 eV can be divided into two peaks at about 374.2 and 373.4 eV, 367.4, and 368.4 eV, respectively. Two strong peaks at 373.4 and 367.4 eV are attributed to Ag3*d*_3/2_ and Ag 3*d*_5/2_ of AgX in Ag–AgX(X = Cl, Br)@TiO_2_. And the peaks at 374.2 and 368.4 eV are attributed to the metallic Ag of Ag3*d*_3/2_ and Ag 3*d*_5/2_ in Ag–AgX(X = Cl, Br)@TiO_2_ [17]. On the basis of XPS results, it can be confirmed that the coexistence of Ag(0) and AgX on the surface of TiO_2_. EDS results are consistent with those of XPS, demonstrating distribution of Ag, Ti, O, and X (Cl, Br) on the samples as shown in the inset in Fig. 4a, b.

The element of Cl and Br comes from C_16_H_33_(CH_3_)_3_NCl (CTAC) and C_16_H_33_(CH_3_)_3_NBr (CTAB), respectively. Their long molecular chain, about 2.3–2.5 nm, is favorable to connect with TiO_2_, but is not in tangle. On the basis of the above experimental results, a possible two-step growth mechanism for the formation of Ag–AgX(X = Cl, Br)@TiO_2_ NPAS is proposed. In the first step, homogenously distributed AgX(X = Cl, Br) nanoparticles on the surface of TiO_2_-aggregated sphere are formed via an interaction between CTAC or CTAB and AgNO_3_, which induces nanoparticles to assemble into the final AgX(X = Cl, Br)@TiO_2_ microspheres. The microspheres of Ag–AgX(X = Cl, Br)@TiO_2_ are formed in the second step when they are exposed to the simulated sunlight. Unbound metallic Ag (Ag^0^) appears due to the photo-induced reduction reaction of AgX nanoparticles. Free metallic Ag(Ag^0^) attaches each other on the surfaces of AgX(X = Cl, Br)@TiO_2_ and forms the final Ag–AgX(X = Cl, Br)@TiO_2_ NPAS. The reduced Ag^0^ atoms may also react with other silver species (Ag^+^ ions, Ag^0^ atoms, or silver clusters). The formation process is shown by the following equations [30]:

1 $$ {\text{AgCl}}{\xrightarrow{{{\text{hv}}}}}{\text{Ag}}^{0} {\text{  +  Cl}}^{0}$$

2 $${\text{AgBr}}{\xrightarrow{{{\text{hv}}}}}{\text{Ag}}^{0} {\text{  +  Br}}^{0} $$

3 $$ {\text{nAg}}^{ 0} \to ( {\text{Ag}}^{ 0} )_{\text{n}} $$

4 $$ {\text{Ag}}^{ 0} + {\text{Ag}}_{\text{r}}^{\text{q + }} \to {\text{Ag}}_{{ ( {\text{r + 1)}}}}^{\text{q + }} \quad \left( {{\text{r}} \ge 1 ; 0\le {\text{q}} \le {\text{r}}} \right) $$

**Fig. 5:**
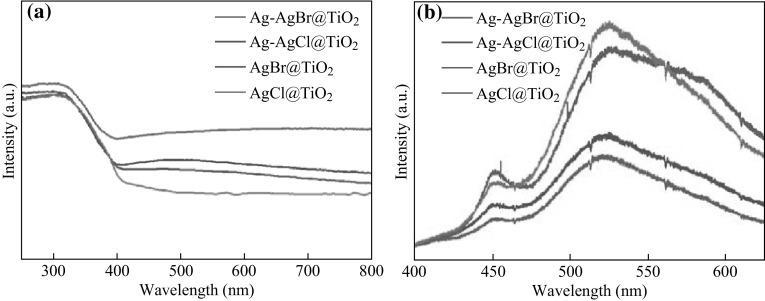
**a** UV–Vis absorption spectra and **b** PL spectra of Ag–AgCl@TiO_2,_ Ag–AgBr@TiO_2_, AgCl@TiO_2_ and AgBr@TiO_2_ structures

To understand the formation of Ag clusters and its role in photoactivity in Ag–AgX@TiO_2_ NPAS, we prepared the AgX@TiO_2_ NPAS in the same way without irradiation. Figure 5a displays the UV–Vis absorption spectra of Ag–AgCl@TiO_2_, Ag–AgBr@TiO_2_, AgBr@TiO_2_, and AgCl@TiO_2_. It is well known that the band gap of anatase TiO_2_ is about 3.2 eV. However, the absorption edge of the four samples is around 410 nm (about 3.1 eV), which is smaller than the band gap of anatase TiO_2_. It might result from surface defect of nanoparticle aggregation spheres with large specific surface and imprecise measurement, which contribute to the absorption edge starting at a little larger wavelength [31]. In addition, AgCl and AgBr on the surface of TiO_2_ have little influence on the absorption edge of Ag–AgX@TiO_2_ NPAS. AgCl@TiO_2_ has no absorption in visible light because of the large band gap of AgCl (about 3.3 eV), while AgBr@TiO_2_ has weak absorption in the visible light due to its smaller band gap (about 2.6 eV). Obviously, Ag–AgCl@TiO_2_ has weak visible-light absorption. Thus, the photoactivity of Ag–AgCl@TiO_2_ extends from the UV into the visible-light region. The absorption of Ag–AgBr@TiO_2_ in the range of 400–800 nm is stronger than that of AgBr@TiO_2_ because of the emergence of Ag. Therefore, as the accumulated excited electrons on the surface of AgX combine with Ag^+^ to form Ag^0^ clusters under the simulated sunlight irradiation, Ag–AgX@TiO_2_ is formed. Silver clusters have empty energy levels below the conduction band of AgCl, which enables a new electronic transition from the valence band of AgCl to the empty Ag cluster energy levels [30]. This is the well-known self-photosensitive property of AgX semiconductor, and it extends the light absorption from the UV to the visible region because of the silver clusters adsorbed on AgCl@TiO_2_ surface [30]. The self-sensitization is different from the spectral sensitization effect of Ag clusters that makes an electron injection from Ag clusters into the AgCl conduction band [19]. Thus, the absorption in the visible light comes from the combined action of the surface plasmon resonance of metallic Ag and the self-sensitization effect of Ag clusters.

Silver clusters have contributed not only to light absorption but also the separation of the excited carriers. The photoluminescence (PL) emission spectroscopy provides the separation efficiency of the electron–hole pairs and carrier lifetime in semiconductors [32]. The PL spectra of Ag–AgCl@TiO_2_, Ag–AgBr@TiO_2_, AgBr@TiO_2_, and AgCl@TiO_2_ (Fig. 5b) show two characteristic bands with peaks at 451 and 523 nm caused by the e–h recombination with 350 nm excitation, which correspond to the emission of TiO_2_ [33]. The PL spectra are much similar for the four samples except for the strength. The PL intensity of Ag–AgX@TiO_2_ shows a notable decrease compared with that of AgX@TiO_2_. Thus, the decrease in the PL intensity suggests that Ag–AgX@TiO_2_ NPAS possess a longer carrier lifetime, which demonstrates the enhancement of the photoinduced electron/hole pair separation. It is responsible for the enhanced photocatalytic activity for the pollutant degradation.

### Visible-Light Photocatalytic Activity

**Fig. 6:**
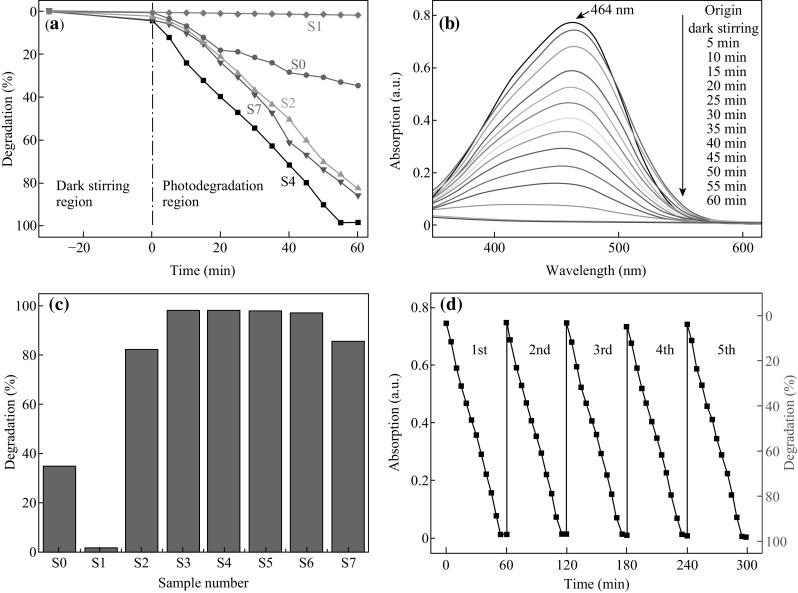
**a** Comparisons of degradation rate with different samples in different stages; **b** absorption spectrums of the degradation with sample S4 in different stages; **c** comparisons of photocatalytic activities among the Ag–AgCl@TiO_2_ NPAS samples with different *R* under the visible light irradiation and TiO_2_ NPAS (S0) sample under the simulated sunlight irradiation in 60 min; **d** The result of the repeated experiments for five times with sample S4

**Fig. 7:**
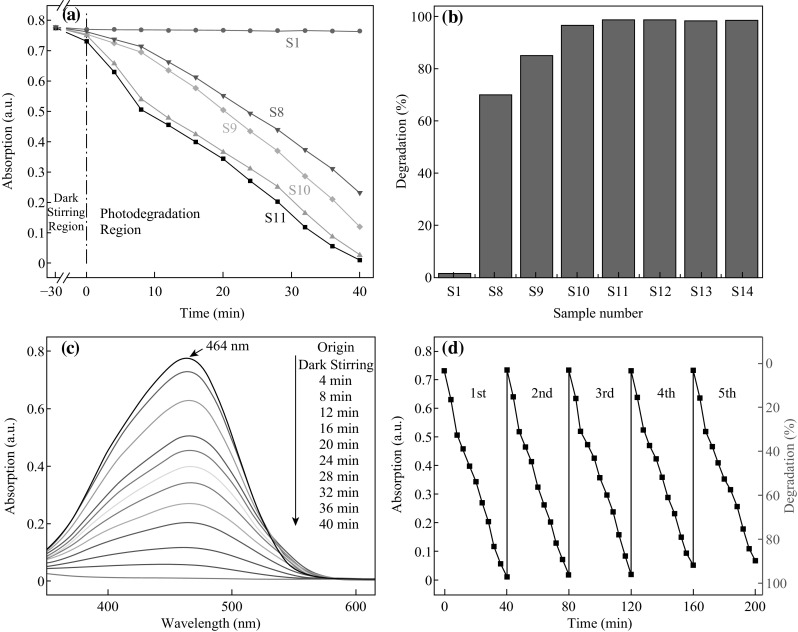
**a** Comparisons of degradation rate with different samples in different stages; **b** comparisons of photocatalytic activities among the Ag–AgBr@TiO_2_ NPAS samples with different R and TiO_2_ NPAS sample under the visible light irradiation in 40 min; **c** absorption spectrums of the degradation with sample S11 in different stages; **d** the result of the repeated experiments for five times with sample S11

MO is a kind of organic dye that is often used as a model pollutant to study the catalytic performance of photocatalysts. In this study, the photocatalytic activity of 20 mg Ag–AgX(X = Cl, Br)@TiO_2_ is assessed by observing the degradation of MO (14 mg L^−1^) versus time under visible-light illumination (Figs. 6, 7). Before irradiation, the MO solution with the catalyst was kept in the dark for 30 min to reach the adsorption equilibrium. The concentration of the MO solution slightly decreases in the dark. Comparisons of photocatalytic activities among the Ag–AgCl@TiO_2_ samples with different *R* under 60-min irradiation are shown in Fig. 6a, b. The degradation activity of sample S1 (TiO_2_ NPAS) is hardly observed due to the larger band gap (*E*_g_ = 3.2 eV). But for the samples S2, S4, and S7, the corresponding degradation rates are 82%, 98%, and 85% under visible-light irradiation, which is much higher than that of S0 (TiO_2_ NPAS under simulated sunlight, only 34.79%). It indicates that greatly improved photocatalytic activity is achieved by the heterostructure of Ag–AgCl@TiO_2_, and the S4 with R = 20:1 exhibits highest activity for MO degradation as shown in Fig. 6a. The temporal evolution of the absorption spectra of MO aqueous solution in presence of S4 under visible-light irradiation is shown in Fig. 6b. The characteristic peaks centered at 464 nm do not change in position in degradation process. It indicates complete photocatalytic degradation of MO aqueous solution by the Ag–AgCl@TiO_2_ NPAS during the reaction, and the catalyzed degradation intermediates are almost not produced. Histogram of photocatalytic activities of Ag–AgCl@TiO_2_ NPAS with different R under visible-light irradiation for 60 min is shown in Fig. 6c. The samples with a wide range R from 10:1 to 25:1 exhibit higher activity for MO degradation with a degradation rate about 98%, which indicates a wide R range for application. It is important to be stable under repeated applications for an economical photocatalyst. Figure 6d shows the cycling performance of sample S4 for five cycles. The photocatalytic activity remains approximately 98%, revealing the excellent long-term photocatalytic stability of Ag–AgCl@TiO_2_ NPAS.

Figure 7 exhibits the activity of Ag–AgBr@TiO_2_ NPAS for MO degradation. It can be seen the efficient removal ratio of 98.78% (for sample S11) in 40 min, which is also far superior to those of Ag–AgCl@TiO_2_ NPAS. Compared with Ag–AgCl@TiO_2_ NPAS, Ag–AgBr@TiO_2_ NPAS have an almost complete photocatalytic degradation of MO and a wider R range from 25:1 to 5:1. Ever after five cycles, there is only about 7.25% of photocatalytic activity decrease for S11, indicating that Ag–AgBr@TiO_2_ NPAS have excellent cycling photocatalytic activity.

### Visible-Light Photocatalytic Mechanism of Ag–AgCl@TiO_2_

**Fig. 8:**
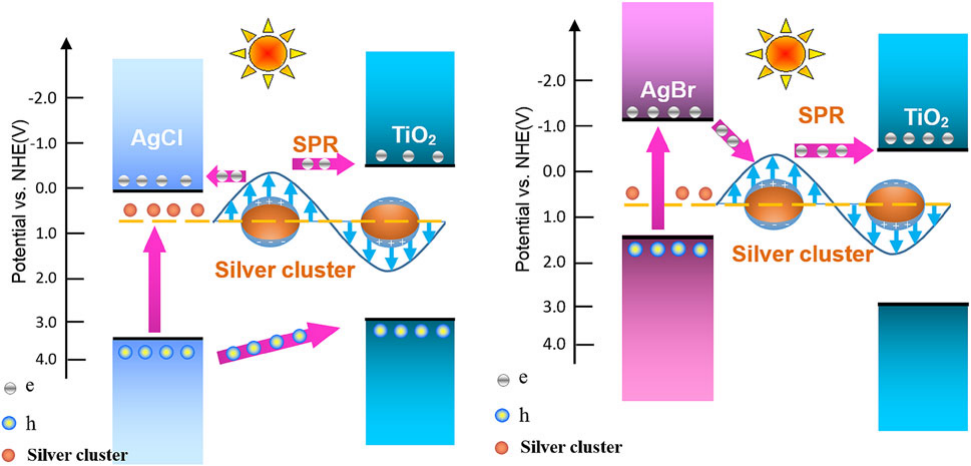
Band structures and degradation mechanisms of **a** Ag–AgCl@TiO_2_ NPAS and **b** Ag–AgBr@TiO_2_ NPAS under visible light

To fully discuss the effects of the heterostructure on the photocatalytic activities of Ag–AgX(X = Cl, Br)@TiO_2_ NPAS, the energy band diagram of Ag, AgX, and TiO_2_ is presented in Fig. 8. AgCl has a direct band gap of 5.6 eV and an indirect band gap of 3.25 eV [21], and the band gap of AgBr is about 2.6 eV. Meanwhile, Ag–AgCl@TiO_2_ NPAS can be excited by visible light as shown in Fig. 5a and it has self-photosensitive effect because of their point ionic defects and electron traps according to the Eqs. (1–5) [13, 34], which might help to photochemical decomposition. It is just similar to the research by Hu group that two electrons in one oxygen vacancy in CeO_2_ are, respectively, excited to two Ce atoms neighboring the vacancy [35]. However, some researchers proposed that plasma resonance absorption of metallic Ag (or Pd) on the surface of semiconductor contributes to the effective visible absorption and segregation of electron/hole pairs, which results in the higher photocatalytic activity [9, 11, 12]. Liu et al. found that the double heterostructure Ag/TiO_2_ nanoparticles/TiO_2_ nanobelts have perfect photocatalytic properties under UV light irradiation with the dominant wavelength of 355 nm and Ag nanoparticles on the surface of the TiO_2_ act as a sink for electrons contributing to the interfacial charge–transfer separation [36]. However, the composite photocatalyst with a wide weight ratio of TiO_2_/Ag_2_O exhibits a higher photocatalytic activity, but a bad cyclic stability under UV light irradiation because Ag^+^ is reduced to Ag [37]. Therefore, the role of Ag on the photocatalytic performance in Ag–AgCl@TiO_2_ NPAS is not determined.

Thus, it is fascinating to consider how Ag/AgCl particles become an efficient and stable photocatalyst under visible light. To fully understand the photocatalysis process and the role of Ag nanoparticles during photocatalytic degradation MO, the XPS spectrum of the Ag–AgCl@TiO_2_ after five cycles is presented in Fig. 4h. The two strong peaks at 373.4 and 367.4 eV corresponding to Ag3*d*_3/2_ and Ag 3*d*_5/2_ of AgCl in Ag/AgCl @TiO_2_ become stronger, while the peaks at 374.2 and 368.4 eV corresponding to the metallic Ag of Ag 3*d*_3/2_ and Ag 3*d*_5/2_ become weaker. It indicates that some metallic Ag are oxidized to Ag^+^ and the self-sensitized effect of AgCl is suppressed during the photocatalysis reaction. Meanwhile, the XPS analysis and the photocatalysis under visible light prove that AgCl is stable at the photocatalytic degradation MO.

Therefore, the photocatalytic process of Ag–AgCl/TiO_2_ under the visible light is as following. Firstly, under visible light irradiation, electrons on valence band of AgCl can be excited to empty silver cluster energy levels below the conduction band of AgCl and form photogenerated electron–hole pairs due to the energy of light lower than the band gap of AgCl [30]. Subsequently, because the CB bottom (0.11 eV vs. NHE) and VB top (3.04 eV vs. NHE) of AgCl are below that of TiO_2_ (*E*_CB_ = −0.41 eV, *E*_VB_ = 2.37 eV), respectively [17], most of excited electrons coming from the surface plasmonic resonance of Ag cluster transfer to the conduction band AgCl and some excited electrons reach to the CB of TiO_2_. The excited electrons react with O_2_ adsorbed on the surface of the Ag–AgCl@TiO_2_ NPAS to produce ·O_2_^−^ and H_2_O_2_ that successively decompose the MO to the final products. Holes transferring to the AgCl surface oxidize Cl^−^ ions to ·Cl radicals, which recombine very fast to form Cl_2_, and then Cl_2_ is reduced to chloride ions by reacting with H_2_O under the applied conditions [13, 30]. Silver cations act as a catalyst for the decomposition of hypochloric acid into molecular oxygen, protons, and chloride ions. Hypochloric acid can also degrade the MO into micromolecule. Meanwhile, some holes also move to the valence band of TiO_2_. The holes on the surface of TiO_2_ can also oxidize OH^−^ to yield $^{{ \cdot }} {\text{OH}}$. Therefore, the degradation of MO could be attributed to the reaction with $^{{ \cdot }} {\text{O}}_{2}^{\text{ - }}$,$^{{ \cdot }} {\text{OH}}$, and H_2_O_2_ species during the photocatalysis. The excellent photocatalytic performance of Ag–AgCl@TiO_2_ NPAS could be due largely to the improvement of electron/hole pairs separation after loading Ag–AgCl on the TiO_2_. The reaction can be written as follows [17, 30],

5 $$  {\text{AgCl(Ag}}_{{\text{s}}}^{{\text{0}}} ){\xrightarrow{{{\text{hv}}}}}({\text{AgCl}})\left[ {{\text{Ag}}_{{\text{s}}}^{ - } ,{\text{Ag}}_{{\text{s}}}^{{\text{0}}} } \right] + {^{ \cdot } {\text{Cl}}} $$

6 $$ 2 ^ { \cdot }{\text{Cl}} \to {\text{Cl}}_{ 2} $$

7 $$ {\text{Cl}}_{ 2} {\text{ + H}}_{ 2} {\text{O}} \to {\text{HOCl + H}}^{ + } {\text{ + Cl}}^{\text{ - }} $$

8 $$ 2{\text{HOCl}}{\xrightarrow{{{\text{Ag}}}}}2{\text{H}}^{ + } {\text{  +  2Cl}}^{{{\text{  -  }}}} {\text{  +  O}}_{2}   $$

9 $$ {\text{Ag}}_{\text{s}}^{\text{ - }} {\text{ + O}}_{ 2} \to  {^ { \cdot }{\text{O}}_{ 2}} {\text{ + Ag}} $$

10 $$ {\text{AgCl(h}}^{ + } ) {\text{ + H}}_{ 2} {\text{O}} \to {\text{AgCl + }} ^ { \cdot }{\text{OH + H}}^{ + } $$

11 $$ {\text{AgCl(e}}^{\text{ - }} ) {\text{ + O}}_{ 2} \to {\text{AgCl + }}^{ \cdot }{\text{O}}_{ 2}^{\text{ - }} $$

12 $$ {\text{TiO}}_{ 2} ( {\text{e}}^{\text{ - }} ) {\text{ + O}}_{ 2} \to {^{ \cdot }{\text{O}}_{ 2}^{\text{ - }}} {\text{ + TiO}}_{ 2} $$

13 $$ {\text{TiO}}_{2} ({\text{h}}^{ + } ){\text{  +  H}}_{2} {\text{O}} \to {\text{TiO}}_{2}  + {^{ \cdot }} {\text{OH  +  H}}^{ + } $$

Therefore, chlorine and hypochloric acid are inevitable during the photocatalytic reaction. Combining Eqs. (1) and (5–13), it indicates the excellent stability of Ag–AgCl@TiO_2_ NPAS photocatalyst in the photocatalytic reaction under visible-light irradiation. Furthermore, it is well known that the Ag nanoparticles are likely to be slowly oxidized in the moist environment [13, 30]. Therefore, the XPS peaks of Ag^+^ become stronger after photocatalytic reaction.

In summary, as a promising candidate, Ag–AgCl@TiO_2_ photocatalyst shows highly efficient and stable photocatalytic activities under visible light. Ag clusters play a significant role in the light harvesting, and the double heterostructure also plays a role in the prevention of the recombination of photogenerated electron–hole pairs.

### Visible-Light Photocatalytic Mechanism of Ag–AgBr@TiO_2_

Figure 8b shows the band structure of Ag, AgBr [16, 18, 19, 38], and TiO_2_, which indicates the photocatalytic mechanism of the Ag–AgBr@TiO_2_ photocatalyst. Under visible-light irradiation, electrons in AgBr (*E*_CB_ = −0.67 and *E*_VB_ = 1.93 eV) are excited from VB to CB for the narrow band gap of 2.6 eV and the photogenerated electrons easily transfer into metal Ag nanoparticles because the CB potential of AgBr is more negative than that Fermi level of the loaded metal Ag. Then some of the electrons in Ag from the AgBr transfer to the conduction band of TiO_2_ (*E*_CB_ = −0.41 eV, *E*_VB_ = 2.37 eV) with the help of the Ag plasma resonance. The charge transfer process of AgBr → Ag → TiO_2_ is similar to the efficient photosynthesis with charge flow steering [38–40]. The Ag clusters in the double heterojunction system act as a charge mediator for transferring electrons to the conduction band of TiO_2_ and keeping holes in the valence band of AgBr. The stronger photoresponse in visible region and efficient carrier separation of Ag–AgBr@TiO_2_ finally enhance the photocatalytic activity more than that of Ag–AgCl@TiO_2_.

Since the XPS spectra of Br 3*d*_5/2_ and Br 3*d*_3/2_ in Ag–AgBr@TiO_2_ photocatalyst become weak after five cycles of photocatalytic degradation MO as shown in Fig. 4i, AgBr in Ag–AgBr@TiO_2_ NPAS is resolved into element Br and element Ag during the reaction of photocatalytic degradation MO according to the Eq. (2), which results in the decrease of Ag–AgBr@TiO_2_ NPAS on photocatalytic activity in degradation MO. However, the decomposition of AgBr does not obviously affect the photocatalytic activity, especially at the first three cycles because of the photocatalytic degradation of MO. Ag–AgBr@TiO_2_ NPAS are excellent in a wider range ratio (*R* = TiO_2_/AgBr) from 25:1 to 5:1, and then the stability of photocatalytic activity is gradually worse until R is larger than 25:1. The photocatalytic reaction of Ag–AgBr@TiO_2_ agrees with the report of Yue group [38].

Owing to the self-sensitization, AgBr is easily decomposed into Ag and Br under visible-light irradiation. As a strong oxidant, Br photodegrades MO into CO_2_ and H_2_O. As a self-sacrificing of AgBr in Ag–AgBr@TiO_2_ photocatalyst, the photodegradation of MO with Ag–AgBr@TiO_2_ is similar to the Ag_2_O/TiO_2_ nanobelts reported by Liu et al. [25]. Therefore, the stability of Ag–AgBr@TiO_2_ photocatalyst is slightly worse than that of Ag–AgCl@TiO_2._

## Conclusions

In this paper, Ag–AgX(X = Cl, Br)@TiO_2_ NPAS synthesized by a facile sol–gel technique and post-photoreduction method. On the basis of their efficient and stable photocatalytic activities, these photocatalysts could be widely used for environmental purification of organic pollutants in aqueous solution. As a self-sensitization material, both AgCl and AgBr have perfect photoresponse in visible light, especially AgBr. Both of them can efficiently separate excited electrons and holes via the fast electron transfer to metal Ag nanoparticles on the surface of heterostructure. The photocatalytic activity of Ag–AgBr@TiO_2_ is higher than Ag–AgCl@TiO_2_ at the first stage. This work indicates that, as an electronic transmission medium, metallic Ag can enhance photocatalytic activity not only via forming heterostructure with narrow band gap photocatalysts (e.g. *E*_g_ = 2.6 eV) but also for wide band gap photocatalysts (e.g. *E*_g_ = 3.2 eV) through the different roles. Ag nanoparticles on the surface of AgCl can effectively help to absorb visible light by self-sensitization and SPR and maintain stability of AgCl, while Ag nanoparticles in Ag–AgBr@TiO_2_ system act as a charge mediator for transferring electrons from AgBr to TiO_2_.

## Electronic supplementary material

Below is the link to the electronic supplementary material.

Supplementary material 1 (PDF 428 kb)
