# Supplementary material for: Enhanced Photocatalytic Activity of Nanoparticle-Aggregated Ag–AgX(X = Cl, Br)@TiO2 Microspheres Under Visible Light
Source: Nanomicro Lett. 2017 Jul 19;9(4):49. doi: 10.1007/s40820-017-0150-8 (PMC6199042; doi:10.1007/s40820-017-0150-8)
Supplement: Supplementary file 1 — Supplementary material 1 (PDF 428 kb) [file 40820_2017_150_MOESM1_ESM.pdf]

Supporting Information for

Enhanced Photocatalytic Activity of Nanoparticle-Aggregated Ag-AgX(X=Cl, Br)@TiO_2 Microspheres Under Visible Light

Cuiling Zhang[1,2,]*, Hao Hua[2], Jianlin Liu[2], Xiangyu Han[2], Qipeng Liu[2], Zidong Wei[2], Chengbin Shao[1], Chenguo Hu[2,]*

[1]Chongqing Engineering Laboratory for Detection, Control and Integrated System, Chongqing Technology and Business University, Chongqing 400067, People's Republic of China

[2]College of Chemistry and Chemical Engineering, College of Physics, Chongqing University, Chongqing 400044, People's Republic of China

*Corresponding authors. E-mail: zhangcl@ctbu.edu.cn, hucg@cqu.edu.cn

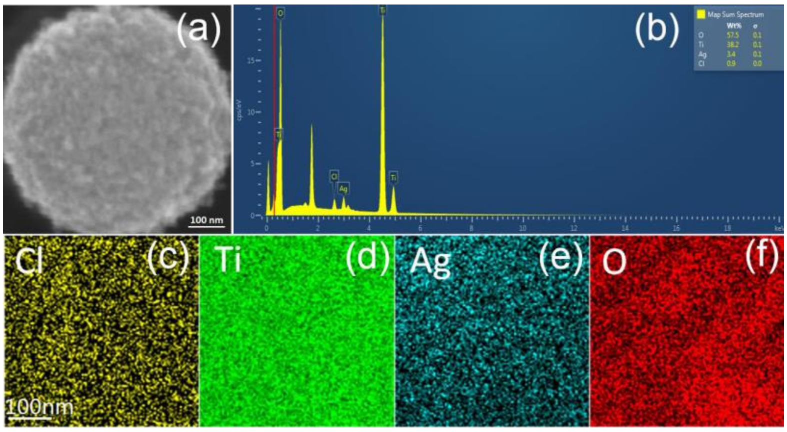

Fig. S1 SEM and EMIs of Ag-AgCl@TiO_2 NPAS

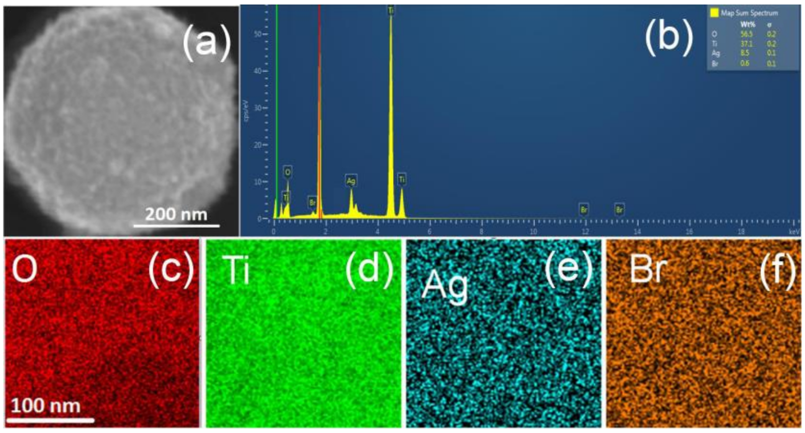

Fig. S2 SEM and EMIs of Ag-AgBr@TiO_2 NPAS
